# Comparative study of plasma microbial cell-free DNA sequencing to culture and polymerase chain reaction in pediatric community-acquired pneumonia with parapneumonic effusion or empyema

**DOI:** 10.1128/jcm.01216-25

**Published:** 2026-02-13

**Authors:** Erin C. Ho, Yuanqing Liu, Kaitlin E. Olson, Edwin J. Asturias, Molly Butler, Dennis Simmons, Samuel R. Dominguez

**Affiliations:** 1 Department of Pediatrics, University of Colorado School of Medicine 12225, Aurora, Colorado, USA; 2 Section of Infectious Diseases and Epidemiology, University of Colorado School of Medicine 12225, Aurora, Colorado, USA; 3 Department of Biostatistics and Informatics, University of Colorado Denver 12226, Aurora, Colorado, USA; 4 Department of Pathology and Laboratory Medicine, Children’s Hospital Colorado, Aurora, Colorado, USA; National Institute of Allergy and Infectious Diseases Division of Intramural Research, Bethesda, Maryland, USA

**Keywords:** pediatrics, complicated community-acquired pneumonia, plasma microbial cell-free DNA sequencing, immunocompetent host

## Abstract

Plasma microbial cell-free DNA (mcfDNA) sequencing is a novel diagnostic tool for pediatric complicated community-acquired pneumonia (cCAP). However, rigorous evaluation of real-world mcfDNA performance is lacking. We compared mcfDNA sequencing to a composite reference standard consisting of blood cultures, pleural fluid (PF) cultures, and targeted PF PCRs in children with cCAP requiring pleural effusion or empyema drainage at Children’s Hospital Colorado from 2022 to 2024. We calculated positive/negative percent agreement (PPA/NPA), Jaccard similarity index, and theoretical time to pathogen detection. We investigated mcfDNA positivity in pediatric controls without bacterial infections. Receiver operating characteristic (ROC) analysis explored potential mcfDNA detection cutoff values to define “true clinical positivity.” Across 45 cCAP cases, mcfDNA sequencing detected a probable pathogen in 86.7% versus 8.9% by blood culture, 20.0% by PF culture, and 71.1% by PF PCRs (*P* < 0.001). Against the culture+PCR composite reference standard, mcfDNA had a PPA of 91.9% (83.1%–100.0%), NPA of 35.7% (10.6%–60.8%), and a Jaccard index of 0.74. Bacterial cfDNA was detected in 52% of controls. ROC analysis yielded an area under the curve of 0.9, with potential optimal detection cutoffs ranging between 158 and 491 mcfDNA molecules per microliter. Theoretical median time to pathogen detection was 3.0 days with mcfDNA versus 4.5 days for pleural fluid PCRs, driven primarily by PF sampling time. Plasma mcfDNA sequencing had a significantly higher diagnostic yield than cultures and a similar yield to PF PCRs; however, over half of non-bacterial controls had low-level mcfDNA detected, potentially complicating interpretation. mcfDNA detection level cutoffs may help elucidate the clinical significance of detected pathogens.

**IMPORTANCE:**

More sensitive diagnostic tests, particularly non-invasive options, are needed to better identify the causative organism(s) in children with complicated community-acquired pneumonia and help inform pathogen-directed therapy. A novel, potentially powerful diagnostic tool for pneumonia is plasma microbial cell-free DNA sequencing, available commercially as Karius Spectrum. However, unknowns regarding its real-world performance and proper role in clinical practice remain. This study aims to address two ongoing concerns: first, the lack of robust comparisons of microbial cell-free DNA (mcfDNA) sequencing performance against validated conventional and state-of-the-art diagnostic modalities (i.e., pleural fluid testing and blood cultures); and second, the unknown baseline positivity rates of mcfDNA in children without bacterial infections. Results from this study may help inform clinical practice decisions and testing implementation strategies.

## INTRODUCTION

Plasma microbial cell-free DNA (mcfDNA) sequencing is a novel, non-invasive diagnostic tool for pediatric complicated community-acquired pneumonia (cCAP). It is available commercially as Karius Spectrum with a turnaround time of 63 h from sample collection to result report (1) and can simultaneously evaluate for over 1,000 microbes in a single blood sample (2). Although retrospective studies have shown promise for its use in pediatric cCAP (3, 4), rigorous studies comparing the real-world performance of mcfDNA against other conventional and state-of-the-art diagnostics are lacking; as such, uncertainties remain regarding in which patients and which clinical scenarios mcfDNA sequencing should be performed.

The baseline positivity of background mcfDNA in uninfected children is also largely unknown, leading to potential misinterpretation of the pathogenicity or clinical significance of detected mcfDNA; this, in turn, could result in the wrong diagnosis with unwarranted changes in antimicrobial therapy or unnecessary further investigation. Initial validation studies for Karius Spectrum found that 22.8% of asymptomatic adult controls had circulating non-pathogenic bacterial cfDNA detectable by sequencing, mostly at levels under 300 mcfDNA molecules per microliter (2). To our knowledge, there are no analogous studies with pediatric controls. Given the higher rates in children, compared to adults, of respiratory tract colonization by potentially invasive bacteria, such as *Streptococcus pneumoniae* (5), *Streptococcus pyogenes* (6), *Haemophilus influenzae* (7–9), and *Staphylococcus aureus* (10, 11), understanding whether mcfDNA from these bacteria is detectable in asymptomatic children or those with viral infections is critical to accurate result interpretation, not only for pneumonia diagnosis but across many infectious syndromes.

To fill these gaps, we compared the positive agreement, negative agreement, and similarity of mcfDNA sequencing to other cCAP testing performed in parallel. We also assessed the positivity of mcfDNA using a convenience sample of children without evidence of bacterial infections. Finally, we explored potential improvements in pathogen detection yield and speed if mcfDNA sequencing were performed early in the diagnostic pathway.

## MATERIALS AND METHODS

### Study population

cCAP was defined as clinically suspected CAP with radiographic evidence of parapneumonic effusion, empyema, abscess, or necrosis. Pneumonia cases were children hospitalized for cCAP at Children’s Hospital Colorado (CHCO) from 2022 to 2024 who underwent pleural fluid (PF) drainage and had residual PF and plasma specimens available for testing. We excluded children who developed pneumonia >48 h after hospitalization, those with witnessed preceding aspiration or seizure events, non-infectious effusions (e.g., chylous, heart failure-related), and those with underlying cystic fibrosis or severe neutropenia.

Non-bacterial controls were selected from a convenience sample of residual frozen K2-EDTA plasma specimens from pediatric patients at CHCO inpatient or ambulatory sites between 2022 and 2025 that had originally been collected for prior research or routine clinical care. We reviewed patient notes, as well as laboratory, imaging, and microbiology results from the electronic medical record (EMR), to select plasma specimens from two types of non-bacterial controls: children without any sick symptoms (“asymptomatic control”) and those with symptoms consistent with a viral upper or lower respiratory tract infection (“viral control”) at the time of plasma collection. We excluded samples from children with documentation of any suspected or proven bacterial infections 2 weeks before or after plasma collection, such as those with any clinical, radiographic (including uncomplicated radiographic pneumonia), microbiologic, or laboratory evidence of a bacterial infection, and those who received antibiotics for any reason within that 2-week time frame. We also excluded samples from children with underlying cystic fibrosis, active malignancy, recent surgery, or overt concern for gastrointestinal mucosal impairment (e.g., mucositis, diarrhea).

Patient characteristics, imaging, testing, and treatments were extracted from the EMR. The Colorado Multiple Institutional Review Board approved this study and granted a waiver of consent.

### Diagnostic testing methods

Plasma mcfDNA sequencing (Karius Spectrum) was performed in the Karius, Inc. CLIA-certified/CAP-accredited clinical laboratory (Redwood City, California). Sequencing data were analyzed using the bioinformatic pipeline DC3.16, which reports mcfDNA abundance in units of mcfDNA molecules per microliter (MPM). MPM values range from undetected to >316,000. Samples not meeting quantification quality metrics are reported as “non-quantifiable” (1, 2).

For pneumonia cases, we compared the diagnostic yield of mcfDNA sequencing to blood culture, PF aerobic and anaerobic cultures, and in-house PF PCR assays targeting *Streptococcus pneumonia*e (Spn, laboratory-developed) (12), group A *Streptococcus* (GAS, laboratory-developed) (13), and *Staphylococcus aureus* (*S. aureus*, laboratory-modified Xpert MRSA/SA SSTI PCR, Cepheid, Sunnyvale, California) (13). All cases had plasma specimens collected during hospitalization for mcfDNA sequencing; however, plasma collection timing and whether to send the specimen for real-time commercial testing versus keep the specimen frozen for potential future use were determined by the patient’s treating infectious diseases team (Fig. S1). All cases had PF drained during hospitalization and sent for PF cultures and PF PCRs (any PCRs not ordered for clinical care were later performed on residual samples). All diagnostic specimens were collected within 10 days of initial radiographic cCAP diagnosis.

### Pathogen adjudication and test performance assessment

Detected bacterial pathogens were independently adjudicated by two infectious diseases physicians (EH, SD) as “probable,” “possible,” or “unlikely” pathogens on a case-by-case basis based on their known likelihood of causing cCAP (14, 15) in pneumonia cases and their potential to cause pneumonia (15, 16) in controls. Given the presence of large, unilateral effusion or empyema requiring drainage in all included cases, viral DNA detections by mcfDNA sequencing were determined *a priori* to not be causative of cCAP in this study.

In the absence of a reference gold standard, we compared the yield of mcfDNA sequencing to a composite reference standard consisting of blood cultures, PF cultures, and targeted PF PCRs. We also compared mcfDNA to conventional cultures alone. Following standard definitions, we calculated positive percent agreement (PPA), negative percent agreement (NPA), and Jaccard similarity index (Table S3a). For non-bacterial controls, we calculated mcfDNA positivity for bacterial pathogens with the potential to cause pneumonia.

To address the concern for potential clinically insignificant detections (“clinical false positive”) with mcfDNA sequencing, we assessed mcfDNA positivity in cases and controls at any detectable MPM level, as well as at detection levels >300 MPM (i.e., pathogens detected under 300 MPM were considered negative). This MPM cutoff of 300 was chosen based on the upper end of mcfDNA detection levels in asymptomatic adult controls from Karius validation studies (2). To further explore what detection levels should be considered a “clinical true positive,” a receiver operating characteristic (ROC) curve was constructed. We calculated the area under the curve (AUC) and identified optimal MPM cutoffs to maximize the sum of test sensitivity and specificity (Youden index), and alternatively, maximize sensitivity while maintaining specificity above 80%. Sensitivity and specificity for differentiating between cases and controls were also evaluated at a set cutoff of 300 MPM. Those without a probable pathogen detected had a value assigned of 0 MPM. For simplicity, for those with more than one probable pathogen detected, only the mcfDNA level of the highest quantifiable probable pathogen was included. Pathogen detections with “non-quantifiable” levels were excluded.

### Theoretical impact analysis on pathogen detection speed and yield

Kaplan Meier curves described the time to probable pathogen detection by actual blood and PF cultures versus theoretical PF PCRs if run at the time of PF drainage versus theoretical mcfDNA sequencing if collected and sent for testing at the time of actual blood culture collection (or time of cCAP diagnosis if no blood culture was collected). Based on typical CHCO and Karius laboratory protocols and timelines, we estimated a turnaround time of 6 h for the on-demand *S. aureus* PCR, 24 h for batched GAS and Spn PCRs run daily, and either 48 or 72 h for mcfDNA sequencing, dependent on time of collection and standard laboratory shipping times. Patients without a pathogen detected were censored at hospital discharge.

Patient characteristics were summarized using median and interquartile range (IQR) or frequency and percentage. Cochran’s Q and Binomial Exact tests with Bonferroni adjustment were used to compare differences in diagnostic yield across tests. Confidence intervals for PPA and NPA were calculated using the Wilson score method. The Kruskal-Wallis test and Wilcoxon rank sum test with Bonferroni adjustment were used to compare the maximum probable pathogen detection level across and between cohorts, respectively. Boxplots visually depicted the distribution of MPMs across pathogen types and cohorts. R version 4.4.3 and SAS OnDemand were used for statistical analysis. Significance was set to α = 0.05.

## RESULTS

The median age in our 48-patient cCAP cohort was 5.9 years (IQR: 3.7–10.6), and 54% required critical care. Median days of antibiotic pretreatment prior to PF drainage and mcfDNA specimen collection were 2.2 days (1.1–4.9) and 2.7 days (1.3–6.1), respectively. All cCAP cases had mcfDNA sequencing, PF Spn, GAS, and *S. aureus* PCR testing, and PF culture performed; 93.8% (45/48) had blood cultures. There were 25 controls, including 15 asymptomatic controls and 10 viral controls. The median age of controls was 7.1 years (3.4–8.6) (Table 1).

**TABLE 1:** Patient characteristics for complicated pneumonia cases and controls^*a*^^,*b*^

	cCAP cases (N = 48)	Controls (N = 25)
Age, years	5.9 (3.7, 10.6)	7.1 (3.4, 8.6)
Male sex	20 (42%)	14 (56%)
Underlying medical condition	15 (31%)	15 (60%)
Asthma	7 (15%)	7 (47%)
Neurologic conditions	4 (8%)	2 (8%)
On immunosuppression	0 (0%)	3 (12%)
Congenital heart disease	0 (0%)	2 (8%)
Other	6 (13%)	4 (16%)
PICU-level care	26 (54%)	–^*d*^
Hospital length of stay, days	11.0 (6.9, 14.3)	–
Time from cCAP diagnosis^*c*^ to pleural fluid drainage, days	1.1 (0.6, 2.3)	–
Time from cCAP diagnosis to plasma mcfDNA collection, days	1.6 (0.9, 3.1)	–
Time on antibiotic therapy prior to test/specimen collection, days		–
Blood culture	2.3 (0.2, 4.7)	–
Pleural fluid	2.2 (1.1, 4.9)	–
Plasma mcfDNA sequencing	2.7 (1.3, 6.1)	–
Type of control		
Viral URI/LRTI symptoms at time of testing	–	10 (40%)
Asymptomatic at the time of testing	–	15 (60%)

^*a*^ Values are shown as median (interquartile range) or N (%), where N is the number of patients.

^*b*^ cCAP, complicated community-acquired pneumonia; mcfDNA, microbial cell-free DNA; URI, upper respiratory infection; LRTI, lower respiratory tract infection; PICU, pediatric intensive care unit.

^*c*^ Radiographic diagnosis of cCAP was based on the presence of consolidation with associated moderate or large parapneumonic effusion, necrosis, cavitary lesion, or abscess on chest plain film, ultrasound, or computed tomography.

^*d*^ Not applicable.

Of the 45 cCAP patients with complete blood and PF testing, 86.7% had at least one probable bacterial pathogen identified by mcfDNA sequencing versus 71.1% by targeted PF PCRs, 20.0% by PF culture, and 8.9% by blood culture (*P* < 0.001, Table 2). mcfDNA sequencing detected a total of 43 probable pathogens across 45 patients with a median detection level of 4,047 MPM (IQR: 1,655–13,752, range: 158 to >316,000). Spn (detected in 37.8%, 17/45) and GAS (31.1%, 14/45) were the most frequently detected pathogens. Polymicrobial detections were uncommon by all methods (Table 2). mcfDNA sequencing detected DNA viruses in nine cases, primarily herpes viruses co-detected with bacteria (Table S1). There were no fungal or parasitic detections. Three cCAP patients had mcfDNA detected at non-quantifiable levels (cases 2, 32, and 47, Table S1), and expanded testing for these cases was concordant with mcfDNA results. Notably, case #32 had an adenovirus detected by qualitative nasopharyngeal PCR and quantitative blood PCR with >2,000,000 copies/milliliter. In conjunction with negative cultures and PF PCRs, these findings suggested cCAP secondary to adenovirus infection alone. Most plasma specimens were collected ≤5 days after starting antibiotics, but mcfDNA remained detectable up to 10 days on therapy (Table S1).

**TABLE 2:** Microbiologic findings and test performance, by diagnostic testing modality^*a*^^,^^*b*^

	Total (N = 45^*c*^)	Plasma mcfDNA sequencing (N = 45)	Blood culture (N = 45)	PF culture (N = 45)	PF PCR assays^*d*^ (N = 45)	Cochran’s Q *P* value
Patients with any probable pathogen detected	40 (88.9%)	39 (86.7%)	4 (8.9%)	9 (20%)	32 (71.1%)	<0.001^*g*^
Patients with >1 probable pathogen detected	5 (11.1%)	4 (8.9%)	0 (0%)	1 (2.2%)	0 (0%)	0.02^*h*^
Number of probable pathogens detected across all patients	46	43	4	10	32	–
Patients with specific pathogens detected						
*Streptococcus pneumoniae*	17 (37.8%)	17 (37.8%)	0 (0%)	2 (4.4%)	16 (35.6%)	<0.001^*i*^
Group A *Streptococcus*	14 (31.1%)	14 (31.1%)	2 (4.4%)	3 (6.7%)	14 (31.1%)	<0.001^*j*^
MSSA	2 (4.4%)	2 (4.4%)	1 (2.2%)	1 (2.2%)	2 (4.4%)	0.39
MRSA	0 (0%)	0 (0%)	0 (0%)	0 (0%)	0 (0%)	–
*Haemophilus influenzae*	2 (4.4%)	2 (4.4%)	0 (0%)	0 (0%)	–	0.14
*Streptococcus anginosus* group	5 (11.1%)	4 (8.9%)	0 (0%)	2 (4.4%)	–	0.09
*Fusobacterium* species	2 (4.4%)	2 (4.4%)	0 (0%)	0 (0%)	–	0.14
*Moraxella catarrhalis*	2 (4.4%)	1 (2.2%)	1 (2.2%)	1 (2.2%)	–	1
*Mycoplasma pneumoniae*	1 (2.2%)	1 (2.2%)	0 (0%)	0 (0%)	–	0.37
Number of possible pathogens detected^*e*^	3	2	1	0	0	–
Number of unlikely pathogens detected^*f*^	2	2	1	0	0	–

^*a*^ Values are shown as N (%), where N is the number of patients or the number of pathogens detected.

^*b*^ mcfDNA, microbial cell-free DNA; PF, pleural fluid; No., number; MSSA, methicillin-susceptible *S. aureus*; MRSA, methicillin-resistant *S. aureus*.

^*c*^ 3/48 cases were excluded from the comparison table due to lack of blood cultures.

^*d*^ Pleural fluid PCR assays include species-specific PCRs targeting *Streptococcus pneumoniae*, group A *Streptococcus, *and *S. aureus* (including MRSA).

^*e*^ Possible pneumonia pathogens detected by mcfDNA sequencing included *Leptotrichia wadei *(277 MPM) with all other testing negative in one patient*, Aggregatibacter segnis *(1,537 MPM) in a patient with a polymicrobial infection, and *Streptococcus salivarius *detected only by a single blood culture with Spn detected by mcfDNA sequencing in one patient.

^*f*^ Unlikely pneumonia pathogens included *Staphylococcus epidermidis *detected by blood culture and mcfDNA sequencing (54 MPM) in one patient and *Escherichia coli* detected only by mcfDNA sequencing (101 MPM) with Spn also detected by mcfDNA sequencing in one patient.

^*g*^ Binomial exact test with Bonferroni adjustment: mcfDNA sequencing vs blood culture *P* < 0.001; mcfDNA sequencing vs PF culture *P* < 0.001; mcfDNA sequencing vs PF PCR *P* = 0.094; blood culture vs PF culture *P* = 0.75; blood culture vs PF PCR *P* < 0.001; PF culture vs PF PCR *P* < 0.001.

^*h*^ Binomial exact test with Bonferroni adjustment: mcfDNA sequencing vs blood culture *P* = 0.625; mcfDNA sequencing vs PF culture *P* = 1; mcfDNA sequencing vs PF PCR *P* = 0.625; blood culture vs PF culture *P* = 1; PF culture vs PF PCR *P* = 1.

^*i*^ Binomial exact test with Bonferroni adjustment: mcfDNA sequencing vs blood culture *P* < 0.001; mcfDNA sequencing vs PF culture *P* < 0.001; mcfDNA sequencing vs PF PCR *P* = 1; blood culture vs PF culture *P* = 1; blood culture vs PF PCR *P* < 0.001; PF culture vs PF PCR *P* < 0.001.

^*j*^ Binomial exact test with Bonferroni adjustments: mcfDNA sequencing vs blood culture *P* = 0.002; mcfDNA sequencing vs PF culture *P* = 0.005; blood culture vs PF culture *P* = 1; blood culture vs PF PCR *P* = 0.002; PF culture vs PF PCR *P* = 0.005.

Compared to the culture+PCR composite reference standard, mcfDNA sequencing (any MPM level) had a PPA of 91.9% (95% CI: 83.1%–100.0%), NPA of 35.7% (10.6%–60.8%), and Jaccard index of 0.74 (Table S3b). McfDNA sequencing detected nine additional probable pathogens not detected by the composite reference standard (*Streptococcus intermedius* [3], *Fusobacterium nucleatum* [2], *Haemophilus influenzae* [2], *Mycoplasma pneumoniae* [1], Spn [1]). Compared to just culture-based testing, mcfDNA had a PPA of 75.0% (50.5%–99.5%), NPA of 12.8% (2.3%–23.3%), and a Jaccard index of 0.20 (Table S3c). McfDNA detected 34 additional pathogens not detected by cultures (Spn [15], GAS [10], MSSA [1], *Streptococcus intermedius* [3], *Fusobacterium nucleatum* [2], *Haemophilus influenzae* [2], *Mycoplasma pneumoniae* [1]). There were three pathogens detected by culture that were not detected by mcfDNA (*Streptococcus intermedius* [1], *Prevotella* [1], and *Moraxella catarrhalis* [1]). Test characteristics were similar when considering only mcfDNA detections >300 MPM (Table S3d and e). Among the subset of Spn, GAS, and *S. aureus* detections (*n* = 33), mcfDNA was positive in all instances (with 94% [31/33] of detections >300 MPM), PF PCRs were positive in 97.0% (32/33), and cultures were positive in 21.2% (7/33).

At least one “probable pathogen” (i.e., bacteria known to cause bacterial pneumonia) was detected in 52.0% (13/25) of controls, including 73% (11/15) of asymptomatic controls and 20% (2/10) of viral controls (Table 2). The majority were detected at low MPMs (median: 59 MPM, IQR: 36–176, range: 18–780). At detection levels >300 MPM, mcfDNA from bacteria with potential to cause pneumonia was only detected in 8% of controls. *Haemophilus influenzae* (32.0%, 8/25) and *Moraxella catarrhalis* (20.0%, 5/25) were the most detected bacteria amongst controls; Spn was detected in two samples (Table S2).

**Fig 1:**
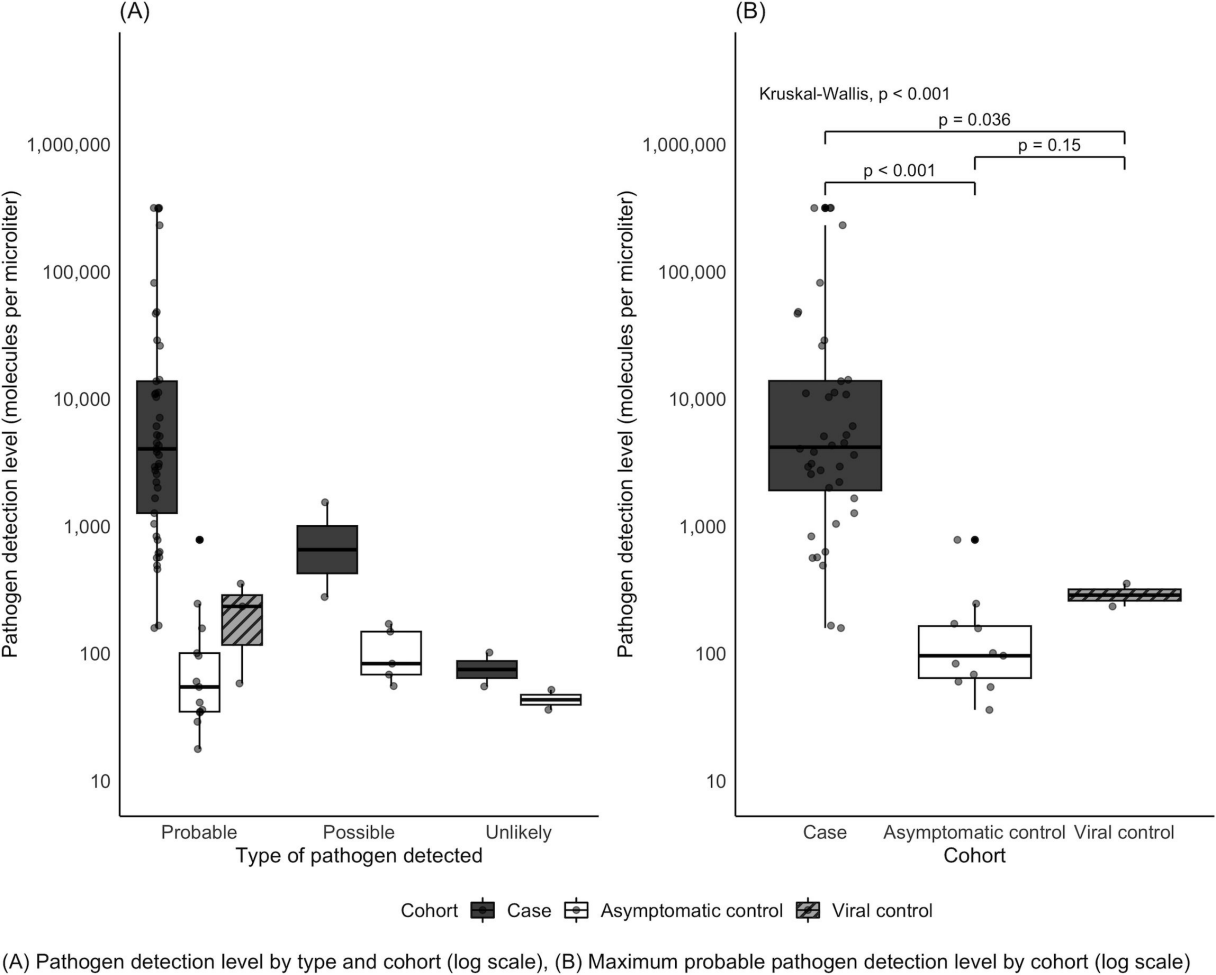
Detection levels of pathogens detected by plasma microbial cell-free DNA sequencing in pneumonia cases and controls (log scale). (**A**) Boxplots comparing pathogen detection levels by type of pathogen detected (probable, possible, unlikely) and by cohort (case, healthy control, viral control). (**B**) Boxplots comparing maximum pathogen detection levels by cohort for detected probable pathogens only. Data points represent the probable pathogen with the highest detection level per patient if more than one probable pathogen is detected.

When considering all pathogen types detected across cases and controls, the MPMs of probable pathogens in cCAP cases were notably higher than in controls (Fig. 1A). Specifically, the median maximum probable pathogen (MPM) was significantly higher for cCAP cases compared to asymptomatic and viral controls (*P* < 0.001, Fig. 1B).

**Fig 2:**
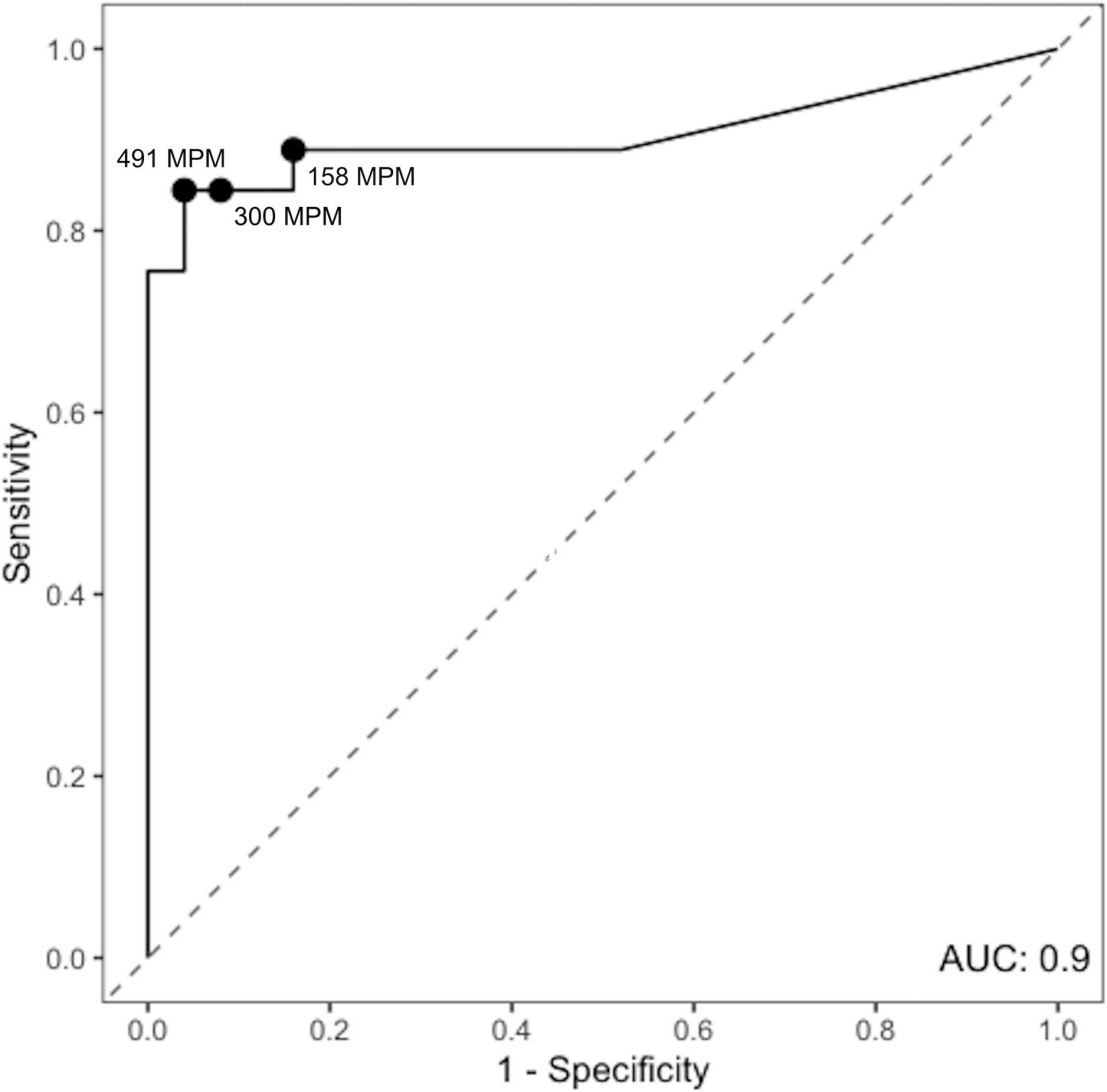
ROC curve describing the performance of plasma mcfDNA sequencing for the diagnosis of complicated pneumonia based on the maximum probable pathogen detection level per patient. N = 70 (three cCAP cases excluded due to “non-quantifiable” pathogen detection levels). AUC is 0.9. The sensitivity and specificity of mcfDNA sequencing to differentiate between cCAP cases and controls at each labeled cutoff point are as follows: at 300 MPM, sensitivity = 84%, specificity = 92%; at 491 MPM, sensitivity = 84%, specificity = 96%; and at 158 MPM, sensitivity = 89%, specificity = 84%. The curve for an arbitrary test that is expected to have no discriminatory value appears as a diagonal line.

ROC analysis included 70 patients (45 cases and 25 controls) and yielded an AUC of 0.9 (Fig. 2). For our cohort, at a detection threshold of 300 MPM, mcfDNA sequencing had a sensitivity of 84% and specificity of 92% for differentiating between cases and controls. Increasing the cutoff to 491 MPM maintained a sensitivity of 84% while increasing specificity to 96%. Conversely, lowering the cutoff to 158 MPM modestly improved sensitivity to 89% but decreased specificity to 84%.

**Fig 3:**
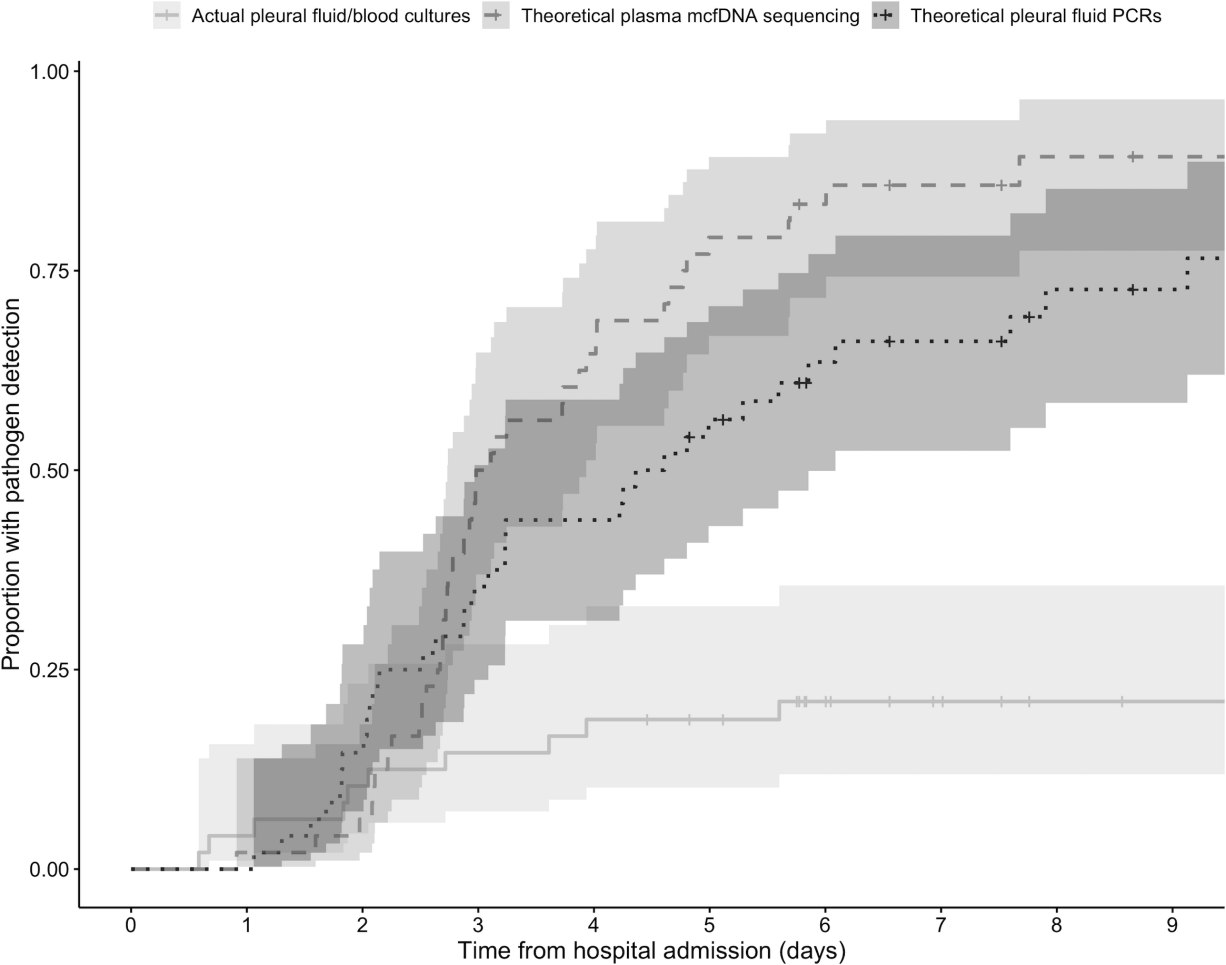
Theoretical impact analysis on time to pathogen detection for cCAP cases by testing modality (N = 48). Kaplan-Meier curves of time from hospital admission to pathogen detection based on the solid line: actual blood and pleural fluid culture testing. Dashed line: theoretical plasma microbial cell-free DNA sequencing if collected and sent for testing at the time of actual blood culture collection (or time of cCAP diagnosis if no blood culture was collected) with a turnaround time of either 48 or 72 h, dependent on collection time. Dotted line: theoretical pleural fluid PCRs if run at the time of pleural fluid drainage, with a turnaround time of 6 h for the on-demand *S. aureus* PCR and 24 h for batched group A *Streptococcus* and *Streptococcus pneumoniae* PCRs run daily. Patients were censored at hospital discharge if they had not achieved the outcome of interest (pathogen detected). Censoring is indicated by vertical tick marks.

Theoretical survival analysis (Fig. 3) demonstrated an increase in both yield and speed of pathogen detection by mcfDNA sequencing and PF PCRs compared to culture. Had mcfDNA sequencing been sent at the time of blood culture collection or initial cCAP diagnosis and all targeted PF PCRs been run at the time of PF drainage, we would expect theoretical median times from hospital admission to pathogen detection to be 3.0 days (95% CI: 2.7–3.9) by mcfDNA sequencing compared to 4.5 days (3.0–6.1) by PF PCR. No median time for cultures is reported, given pathogen yield under 50%.

## DISCUSSION

We found that mcfDNA sequencing detected a probable causative pathogen in over 85% of our cCAP cases, with significantly higher yield than conventional cultures. When combining PF PCRs targeting Spn, GAS, and *S. aureus* to culture-based testing, mcfDNA had a similar yield, with high PPA (91.9%) and acceptable similarity (Jaccard index 0.74) in the pathogens detected across testing methods. The low NPA (35.7%) suggests potential added diagnostic value provided by mcfDNA sequencing, as 9/46 probable pathogens were identified by mcfDNA alone. Notably, mcfDNA of bacterial pathogens with pneumonia potential was detected in over half of our control group of children without evidence of bacterial infection. Most were detected at low levels, and thus, consideration of quantitative detection levels may be helpful in determining the clinical significance of detected pathogens.

In 90% of cCAP cases positive by mcfDNA sequencing, only one bacterial pathogen was detected, which aligns with our epidemiologic understanding of pediatric cCAP as a primarily monomicrobial infection (14, 17). The low percentage with mcfDNA from multiple or unlikely pathogens detected is similar to findings by Dworsky et al. (3). *Streptococcus intermedius* and *Fusobacterium* were co-detected in three cases, highlighting an underrecognized coinfection in pediatric cCAP that has been reported in pediatric and adult pleural infections (3, 18). Molecular detection of *Fusobacterium*, which is difficult to recover by culture, is one prime example of how mcfDNA can facilitate pathogen-directed therapy given robust anaerobic coverage is not empirically recommended by national pediatric CAP guidelines (19). More commonly, with the predominance of Spn and GAS in pediatric cCAP, mcfDNA results may facilitate faster definitive narrowing to ampicillin/amoxicillin, particularly for children with negative conventional testing coupled with illness courses plagued by prolonged fevers, persistently elevated biomarkers, and potential need for multiple procedures to achieve adequate source control. Previously, we found that clinical implementation of Spn PF PCR testing was associated with significantly lower rates of unwarranted broad-spectrum and anti-MRSA coverage and decreased the time to optimal therapy by 5 days, suggesting that knowing the causative pathogen impacts clinician behavior and patient care (20). The impact of mcfDNA may be broader given its pathogen-agnostic approach and ability to perform testing prior to PF drainage, which is often pursued only if and when therapeutically required, but prospective studies are needed to test this hypothesis.

Our simulation decision analysis shows the potential impact that early mcfDNA sequencing could have on pathogen detection, with a median time from hospital admission to etiologic diagnosis of 3 days if sent simultaneously with blood culture collection. The slight improvement in median time to detection for mcfDNA (3.0 days) versus PF PCR (4.5 days) hinges primarily on delays in PF sampling, with time differentials greater for institutions without in-house PF PCR capabilities. A 2–3 day turnaround time for mcfDNA sequencing is likely fast enough to provide clinically actionable results given the median hospital length of stay in our cCAP cohort was 11 days and a typical treatment course for cCAP is around 2–3 weeks, although results from other testing (e.g., positive PF PCRs or cultures, negative MRSA nasal PCR swab) may attenuate actual clinical impact of mcfDNA sequencing.

Our institutional approach to optimize diagnostic yield while promoting high-value care through appropriate diagnostic stewardship is to draw and freeze a plasma sample for mcfDNA sequencing very early in the illness course but wait on results of culture and PCR testing to decide if mcfDNA sequencing is clinically needed (i.e., other testing is negative and patient is not improving or remains on broad-spectrum coverage with resistance to empiric narrowing). Given its excellent negative predictive value in pneumonia, a negative MRSA nasal swab PCR may also be a rapid (if available in-house), cost-effective alternative to mcfDNA sequencing specific to MRSA therapy de-escalation, keeping in mind its limited scope and poor positive predictive value (21–23). For institutions without in-house PCR capabilities or when patients have received significant antibiotic pre-treatment, mcfDNA sequencing may have more up-front utility given the low yield of culture-based testing, albeit currently at a significantly higher cost. As with all new technologies, institutions should consider the comparative cost versus the potential added diagnostic value of each test to inform implementation strategies.

One of the major outstanding concerns with interpreting mcfDNA is detecting potentially clinically insignificant pathogens, leading to poor test specificity, clinical uncertainty, and unnecessary additional antimicrobials or testing (24). Indeed, we found that over half of the children in our control group had bacterial cfDNA detected. We suspect these detections represent asymptomatic colonization, given none of these children required antibiotics or had evidence of any overt bacterial infection, although a mild, self-resolving infection cannot be excluded. Similarly, two recent studies found that children with localized musculoskeletal infections commonly had background *Haemophilus influenzae* cfDNA detected, most of which were discordant with other testing and not thought to be causative of their musculoskeletal infection (25, 26). In contrast, a study of mcfDNA positivity in pediatric cancer patients during afebrile periods (negative controls) found that 18% had bacterial cfDNA detected (27). We query whether frequent antibiotic administration in oncologic patients may explain the lower incidence of background mcfDNA in this study. Interestingly, unlike our controls, most cCAP cases lacked low-level detections of multiple respiratory flora, again possibly due to antibiotic pretreatment for pneumonia prior to mcfDNA specimen collection, although we did not observe more detections of unclear significance in cCAP cases without pretreatment. An alternative hypothesis may be that a very high abundance of cfDNA present from one organism may reduce the likelihood of detection of other microbial cfDNA present at low levels. At our institution, a formal Infectious Diseases consult along with approval by the microbiology laboratory leadership team is required to order mcfDNA sequencing, which we have found to be critically important for proper diagnostic stewardship of mcfDNA sequencing and ensuring appropriately cautious interpretation of results, particularly in cases with mcfDNA results that are discordant with other testing and/or with low-level detections of unclear significance.

Indeed, our results suggest that the detection of commensal, colonizing organisms in healthy children is common. We conclude that quantitative detection level should likely be considered in conjunction with intrinsic organism pathogenicity when interpreting results, and when done so, we found that mcfDNA sequencing can achieve both adequate sensitivity and specificity for the diagnosis of cCAP (ROC curve AUC = 0.9). For our small cohort, the optimal MPM cutoff was likely within the range of 158 to 491 MPM. Future studies with larger sample sizes are needed to further establish potential cut points, ideally stratified by collection timing, pneumonia severity, and specific to the pathogen detected, before it can be applied in widespread clinical practice. Additionally, Karius has recently updated their unit reporting, so additional studies will need to be conducted with these new standards.

Our study had several important limitations. This was a single-center, retrospective study in a region with low MRSA prevalence, possibly limiting generalizability. Our pneumonia cohort included only children with both PF drained and an mcfDNA specimen collected, so findings are not representative of all children hospitalized with cCAP. Due to the retrospective nature of the study, selection of controls was limited to available residual specimens and relied solely on chart review for symptoms and testing performed, so we may have missed mild or undiagnosed bacterial infections. We only included bacterial pathogens as true positives, possibly underestimating diagnostic yield, as one patient likely had severe adenovirus cCAP. Similarly, ROC analysis excluded non-quantifiable detections in three cases, likely underestimating the AUC and sensitivity. Finally, this study focused on comparing the performance of different available testing modalities for cCAP, and due to a significant portion of testing performed retrospectively on residual samples and a small percentage without blood cultures, we were not able to investigate the clinical impact of each modality. Results from this study lay the groundwork for future prospective studies focusing on evaluating the clinical impact of mcfDNA sequencing on treatment decisions, patient outcomes, and cost-effectiveness for pediatric cCAP.

In conclusion, mcfDNA sequencing may be a promising non-invasive testing strategy for cCAP diagnosis. However, cautious test interpretation is required, given the potential detection of non-pathogenic, circulating pathogens.

## Data Availability

The data used to compare the performance of mcfDNA sequencing to other testing and reach the conclusions in this study are included in the supplemental materials as Tables S1 and S2. Additional data is available upon request.

## SUPPLEMENTAL MATERIAL

Supplemental material jcm.01216-25-s0001.docx Figure S1 and Tables S1 to S3.

ASM does not own the copyrights to Supplemental Material that may be linked to, or accessed through, an article. The authors have granted ASM a non-exclusive, world-wide license to publish the Supplemental Material files. Please contact the corresponding author directly for reuse.
