# Supplementary material for: Comparative study of plasma microbial cell-free DNA sequencing to culture and polymerase chain reaction in pediatric community-acquired pneumonia with parapneumonic effusion or empyema
Source: J Clin Microbiol. 2026 Feb 13;64(3):e01216-25. doi: 10.1128/jcm.01216-25 (PMC12977497; doi:10.1128/jcm.01216-25)
Supplement: Supplemental material — Figure S1 and Tables S1 to S3. [file jcm.01216-25-s0001.docx]

**Comparative study of plasma microbial cell-free DNA sequencing to culture and polymerase chain reaction in pediatric community-acquired pneumonia with parapneumonic effusion or empyema**

**Authors:** Erin C Ho, MD, Yuanqing Liu, MA, Kaitlin E Olson, MS, Edwin J Asturias, MD, Molly Butler, PhD, Dennis Simmons, MS, Samuel R Dominguez, MD, PhD

**Supplemental materials**

Supplemental Figure 1: Specimen collection/testing for plasma microbial cell-free DNA sequencing in complicated pneumonia cases

Supplemental Table 1: Microbiologic findings by study participant for complicated pneumonia cases

Supplemental Table 2: Plasma microbial cell-free DNA sequencing results for non-pneumonia control group

Supplemental Table 3 a-e: 2x2 tables comparing plasma microbial cell-free DNA sequencing to reference testing for complicated pneumonia cases

**Supplemental Figure 1:** Specimen collection/testing for plasma microbial cell-free DNA sequencing in complicated community-acquired pneumonia (cCAP) cases

Specimens sent for real-time commercial testing

**(N = 19)**

cCAP patients with plasma specimens collected for mcfDNA sequencing during hospitalization

**(N = 48)**

Specimens frozen for later research testing

**(N = 29)**

Reasons for not sending for commercial testing:

- Positive culture/PCR testing **(N = 21)**
- Patient improving on empiric therapy, other real-time PCR/culture testing negative **(N = 8)**

**Supplemental Table 1: Microbiologic findings by study participant for cCAP cases**

| Study  No. | Age (years) | Month of test collection | Time on antibiotics before PF drainage  (days) | Time on antibiotics before mcfDNA sequencing collection (days) | Plasma microbial cell-free DNA sequencing (molecules per microliter) | Culture results | Pleural fluid  PCR results / Additional molecular testing |
| --- | --- | --- | --- | --- | --- | --- | --- |
| 1 | 17.6 | Jan | 1.5 | 0 | Negative | Blood culture negative  **PF culture +*Streptococcus intermedius/anginosus*** | Spn: ND  MRSA/SA: ND  GAS: ND |
| 2 | 8.0 | Dec | 0.7 | 0.4 | **GAS (not quantifiable)**  Varicella-zoster virus (not quantifiable) | **Blood culture +GAS**  **PF culture +GAS** | Spn: ND  MRSA/SA: ND  **GAS: detected**  *Extra testing: VZV PCR (Diasorin) from skin lesion:* ***detected*** |
| 3 | 9.7 | Dec | 2.1 | 0.2 | **GAS (3,087)** | **Blood culture +GAS**  PF culture negative | Spn: ND  MRSA/SA: ND  **GAS: detected** |
| 4 | 3.6 | Aug | 1.1 | 0.3 | **Spn (>316,000)**  Epstein-Barr virus (133) | **Blood culture +*Moraxella catarrhalis***  **PF culture +Spn** | **Spn: detected**  MRSA/SA: ND  GAS: ND |
| 5 | 6.4 | Dec | 0.6 | 0.9 | *Leptotrichia wadei* (277)  (possible pathogen) | Blood culture negative  PF culture negative | Spn: ND  MRSA/SA: ND  GAS: ND |
| 6 | 13.4 | Mar | 1.8 | 1.0 | Negative | Blood culture negative  PF culture negative | Spn: ND  MRSA/SA: ND  GAS: ND |
| 7 | 2.7 | Apr | 0.5 | 0.6 | **Spn (>316,000)**  ***Moraxella catarrhalis* (311,027)** | Blood culture negative  **PF culture +*Moraxella catarrhalis*** | **Spn: detected**  MRSA/SA: ND  GAS: ND |
| 8 | 10.5 | Jan | 3.4 | 3.5 | **GAS (11,032)** | Blood culture negative  PF culture negative | Spn: ND  MRSA/SA: ND  **GAS: detected** |
| 9 | 11.7 | Apr | 0.6 | 0.5 | ***Streptococcus intermedius* (1,264)**  ***Fusobacterium nucleatum* (778)** | Blood culture negative  PF culture negative | Spn: ND  MRSA/SA: ND  GAS: ND |
| 10 | 8.9 | Mar | 8.0 | 0.8 | **Spn (26,119)**  Human herpesvirus 6B (288) | Blood culture negative  PF culture negative | **Spn: detected**  MRSA/SA: ND  GAS: ND |
| 11 | 1.4 | Nov | 3.2 | 1.1 | **Spn (81,385)** | Blood culture +*Streptococcus salivarius group* (possible pathogen)  PF culture negative | **Spn: detected**  MRSA/SA: ND  GAS: ND |
| 12 | 5.5 | Nov | 5.0 | 2.8 | **MSSA (10,832)**  ***Haemophilus influenzae* (7,107)**  *Agregatibacter segnis* (1,537) (possible pathogen) | **Blood culture +MSSA**  **PF culture +MSSA, *prevotella species*** | Spn: ND  **MRSA/SA: SA detected**, MRSA ND  GAS: ND |
| 13 | 2.1 | Nov | 1.4 | 1.6 | **GAS (13,752)** | Blood culture negative  PF culture negative | Spn: ND  MRSA/SA: ND  **GAS: detected** |
| 14 | 4.1 | Mar | 0.9 | 1.0 | **Spn (231,098)** | Blood culture --  PF culture negative | **Spn: detected**  MRSA/SA: ND  GAS: ND |
| 15 | 4.2 | Jan | 0.9 | 1.4 | **GAS (3,623)** | Blood culture negative  **PF culture +GAS** | Spn: invalid  MRSA/SA: ND  **GAS: detected** |
| 16 | 5.8 | Dec | 7.2 | 7.7 | **Spn (4,496)** | Blood culture negative  PF culture negative | Spn: ND  MRSA/SA: ND  GAS: ND |
| 17 | 8.9 | Jan | 0.1 | 1.5 | **GAS (48,180)** | Blood culture negative  **PF culture +GAS** | Spn: ND  MRSA/SA: ND  **GAS: detected** |
| 18 | 5.8 | Dec | 1.0 | 1.8 | **Spn (2,918)** | Blood culture negative  **PF culture +Spn** | **Spn: detected**  MRSA/SA: ND  GAS: ND |
| 19 | 9.7 | Mar | 2.9 | 3.8 | **Spn (1,655)** | Blood culture negative  PF culture negative | **Spn: detected**  MRSA/SA: ND  GAS: ND |
| 20 | 14 | Mar | 7.8 | 7.8 | **GAS (2,215)** | Blood culture negative  PF culture negative | Spn: ND  MRSA/SA: ND  **GAS: detected** |
| 21 | 6.4 | Jan | 1.0 | 2.0 | **GAS (165)** | Blood culture --  PF culture negative | Spn: ND  MRSA/SA: ND  **GAS: detected** |
| 22 | 1.7 | Mar | 3.7 | 1.9 | **Spn (28,828)** | Blood culture negative  PF culture negative | **Spn: detected**  MRSA/SA: ND  GAS: ND |
| 23 | 2.4 | Aug | 1.9 | 2.2 | **Spn (10,319)**  Human herpesvirus 6B (302) | Blood culture negative  PF culture negative | **Spn: detected**  MRSA/SA: ND  GAS: ND |
| 24 | 14.1 | Nov | 1.5 | 1.5 | *Staphylococcus epidermidis* (55)  (unlikely pathogen) | Blood culture +*Staphylococcus epidermidis*  (unlikely pathogen)  PF culture negative | Spn: ND  MRSA/SA: ND  GAS: ND |
| 25 | 12.5 | May | 0.9 | 2.7 | ***Haemophilus influenzae* (562)**  Epstein-Barr virus (161) | Blood culture negative  PF culture negative | Spn: ND  MRSA/SA: ND  GAS: ND |
| 26 | 6.2 | Mar | 10.7 | 10.7 | **Spn (491)** | Blood culture negative  PF culture negative | **Spn: detected**  MRSA/SA: ND  GAS: ND |
| 27 | 10.6 | Apr | 2.2 | 2.1 | **Spn (4,047)** | Blood culture negative  PF culture negative | **Spn: detected**  MRSA/SA: ND  GAS: ND |
| 28 | 14.7 | Jul | 2.7 | 2.7 | ***Streptococcus intermedius*** **(46,674)** | Blood culture negative  **PF culture +*Streptococcus intermedius/anginosus*** | Spn: ND  MRSA/SA: ND  GAS: ND |
| 29 | 3.7 | Mar | 8.0 | 10.6 | ***Streptococcus intermedius*** **(2,934)** | Blood culture negative  PF culture negative | Spn: ND  MRSA/SA: ND  GAS: ND |
| 30 | 3.9 | Mar | 3.2 | 3.1 | **GAS (>316,000)** | Blood culture negative  PF culture negative | Spn: ND  MRSA/SA: ND  **GAS: detected** |
| 31 | 4.3 | Dec | 1.5 | 3.2 | **GAS (5,086)** | Blood culture negative  PF culture negative | Spn: ND  MRSA/SA: ND  **GAS: detected** |
| 32 | 17.6 | Nov | 5.8 | 7.7 | Human adenovirus B  (not quantifiable) | Blood culture negative  PF culture negative | Spn: ND  MRSA/SA: ND  GAS: ND  *Extra testing: Adenovirus blood PCR (LDT):* ***> 2 million copies/mL****, RPP (BioFire):* ***adenovirus detected;***  PF broad range 16s PCR + sequencing (Mayo): No bacterial DNA detected |
| 33 | 6.2 | Nov | 1.8 | 4.3 | **Spn (2,556)** | Blood culture negative  PF culture negative | **Spn: detected**  MRSA/SA: ND  GAS: ND |
| 34 | 5.5 | Nov | 3.0 | 4.2 | **Spn (11,203)** | Blood culture negative  PF culture negative | **Spn: detected**  MRSA/SA: ND  GAS: ND |
| 35 | 6.9 | Feb | 3.4 | 4.8 | **GAS (14,110)** | Blood culture negative  PF culture negative | Spn: ND  MRSA/SA: ND  **GAS: detected** |
| 36 | 5.8 | Mar | 7.7 | 12.5 | Negative | Blood culture negative  PF culture negative | Spn: ND  MRSA/SA: ND  GAS: ND |
| 37 | 2.6 | Mar | 0.0 | 0.0 | **MSSA (569)** | Blood culture negative  PF culture negative | Spn: ND  **MRSA/SA: SA detected**, MRSA ND  GAS: ND |
| 38 | 3.5 | Nov | 5.2 | 7.4 | **Spn (2,000)**  Epstein-Barr virus (85) | Blood culture negative  PF culture negative | **Spn: detected**  MRSA/SA: ND  GAS: ND |
| 39 | 4.9 | Aug | 0.0 | 0.0 | ***Fusobacterium nucleatum* (830)**  ***Streptococcus intermedius* (458)** | Blood culture negative  PF culture negative | Spn: ND  MRSA/SA: ND  GAS: ND |
| 40 | 2.5 | Jan | 6.3 | 6.4 | **GAS (3,822)**  Epstein-Barr virus (47)  Human adenovirus C (160) | Blood culture negative  PF culture negative | Spn: ND  MRSA/SA: ND  **GAS: detected** |
| 41 | 3.4 | Apr | 4.6 | 7.5 | **Spn (2,738)**  Escherichia coli (101) (unlikely pathogen) | Blood culture negative  PF culture negative | **Spn: detected**  MRSA/SA: ND  GAS: ND |
| 42 | 15.4 | Apr | 2.5 | 6.6 | **GAS (158)** | Blood culture negative  PF culture negative | Spn: ND  MRSA/SA: ND  **GAS: detected** |
| 43 | 4.9 | Apr | 6.7 | 9.7 | **Spn (6,093)** | Blood culture negative  PF culture negative | **Spn: detected**  MRSA/SA: ND  GAS: ND |
| 44 | 2.4 | May | 4.9 | 5.8 | **Spn (5,192)**  Epstein-Barr virus (59) | Blood culture negative  PF culture negative | **Spn: detected**  MRSA/SA: ND  GAS: ND |
| 45 | 3.7 | May | 0.3 | 3.3 | **GAS (627)** | Blood culture negative  PF culture negative | Spn: ND  MRSA/SA: ND  **GAS: detected** |
| 46 | 18.1 | May | 1.7 | 1.9 | **GAS (1,041)** | Blood culture negative  PF culture negative | Spn: ND  MRSA/SA: ND  **GAS: detected** |
| 47 | 6 | Aug | 1.7 | 0.7 | ***Mycoplasma pneumoniae***  **(not quantifiable)** | Blood culture negative  PF culture negative | Spn: ND  MRSA/SA: ND  GAS: ND  *Extra testing: PF Mycoplasma pneumoniae PCR (Mayo):* ***positive;*** *RPP (BioFire):* ***mycoplasma pneumoniae detected*** |
| 48 | 16.6 | Aug | 0.0 | 0.0 | ***Fusobacterium nucleatum* (4,297)**  ***Streptococcus intermedius* (613)** | Blood culture --  PF culture negative | Spn: ND  MRSA/SA: ND  GAS: ND |

**Pathogens in bold** adjudicated to be probable causative pathogen of complicated community acquired pneumonia

-- indicates test not performed

Abbreviations: No., number; cCAP, complicated community-acquired pneumonia; PF, pleural fluid; mcfDNA, microbial cell-free DNA, Spn, *Streptococcus pneumoniae*; GAS, group A *Streptococcus*; SA, *Staphylococcus aureus*; MRSA, methicillin-resistant *Staphylococcus aureus;* LDT, laboratory developed test; RPP, respiratory pathogen panel (nasopharyngeal)

**Supplemental Table 2: Plasma microbial cell-free DNA sequencing results for non-pneumonia control group**

| Study  No. | Age (years) | Month of test collection | Control  type | Plasma microbial cell-free DNA sequencing (molecules per microliter) | At least one pathogen with cCAP potential (a.k.a.,probable pathogen) detected **at any level**? |
| --- | --- | --- | --- | --- | --- |
| 49 | 1.8 | Jun | Viral control | Human bocavirus (1,075) | No |
| 50 | 6.0 | May | Viral control | ***Haemophilus influenzae* (234)** | **Yes** |
| 51 | 7.3 | May | Viral control | Negative | No |
| 52 | 9.0 | Apr | Viral control | Negative | No |
| 53 | 1.2 | Apr | Viral control | Negative | No |
| 54 | 2.2 | Mar | Viral control | Negative | No |
| 55 | 3.4 | Mar | Viral control | Negative | No |
| 56 | 1.8 | Feb | Viral control | Negative | No |
| 57 | 1.3 | Feb | Viral control | ***Haemophilus influenzae* (353)**  ***Moraxella catarrhalis* (58)** | **Yes** |
| 58 | 8.4 | Feb | Viral control | Negative | No |
| 59 | 3.7 | May | Asymptomatic control | ***Streptococcus pneumoniae*** **(96)** | **Yes** |
| 60 | 2.3 | Mar | Asymptomatic control | ***Haemophilus influenzae/haemolyticus* (29)**  ***Streptococcus pneumoniae* (100)**  ***Moraxella catarrhalis* (18)**  Human adenovirus C (39) | **Yes** |
| 61 | 8.1 | Jan | Asymptomatic control | Human herpesvirus 6B (53) | No |
| 62 | 4.4 | Feb | Asymptomatic control | ***Haemophilus influenzae*** (780) | **Yes** |
| 63 | 7.1 | Apr | Asymptomatic control | ***Moraxella catarrhalis*** (60) | **Yes** |
| 64 | 7.4 | Mar | Asymptomatic control | *Bordetella parapertussis* (148) (possible pathogen)  *Enterococcus faecium* (36) (unlikely pathogen) | No |
| 65 | 4.8 | May | Asymptomatic control | ***Haemophilus species*** **(157)**  *Eikenella species* (55) (possible pathogen) | **Yes** |
| 66 | 4.2 | May | Asymptomatic control | ***Moraxella catarrhalis*** **(36)**  Human herpesvirus 7 (13)  Human adenovirus F (9) | **Yes** |
| 67 | 11.8 | Dec | Asymptomatic control | ***Fusobacterium necrophorum* (34)**  *Burkholderia cepacia complex* (170) (possible pathogen) | **Yes** |
| 68 | 14.2 | Dec | Asymptomatic control | ***Haemophilus influenzae* (41)**  *Burkholderia cepacia complex* (83) (possible pathogen) | **Yes** |
| 69 | 13.1 | Dec | Asymptomatic control | ***Haemophilus influenzae* (246)** | **Yes** |
| 70 | 8.6 | Dec | Asymptomatic control | Human herpesvirus 7 (12) | No |
| 71 | 14.0 | Dec | Asymptomatic control | ***Haemophilus influenzae* (35)**  *Burkholderia cepacia complex* (68) (possible pathogen)  *Mycobacteroides immunogenum* (52) (unlikely pathogen) | **Yes** |
| 72 | 10.0 | Dec | Asymptomatic control | Human parvovirus B19 (52) | No |
| 73 | 7.3 | Dec | Asymptomatic control | ***Moraxella catarrhalis* (54)**  Human parvovirus B19 (23)  Human adenovirus C (17) | **Yes** |

**Pathogens in bold** adjudicated to be pathogens with potential to cause complicated community-acquired pneumonia in children (excluding those with severe neutropenia or other specific immunodeficiency increasing risk for CAP from atypical pathogens, cystic fibrosis, or severe underlying lung disease)

**Supplemental Table 3 a-e: 2x2 tables comparing plasma microbial cell-free DNA sequencing to reference testing for complicated pneumonia cases**

**Table 3a: 2x2 template and calculations**

|  |  | **Reference test** | |  |
| --- | --- | --- | --- | --- |
| **Plasma mcfDNA sequencing** |  | **+** | **−** | **Total** |
|  | **+** | a | b | a+b |
|  | **−** | c | d | c+d |
| **Total** |  | a+c | b+d | a+b+c+d |

a = number of probable pathogens detected by both plasma mcfDNA sequencing and reference testing

b = number of probable pathogens detected by only plasma mcfDNA sequencing

c = number of probable pathogens detected by only reference testing

d = number of samples negative by both plasma mcfDNA sequencing and reference testing

**Positive percent agreement** = a/(a+c) x 100%

**Negative percent agreement** = d/(b+d) x 100%

**Jaccard index** = a/(b+c+a) *[(number of pathogens detected by both plasma mcfDNA sequencing and reference test) / (number of pathogens detected by mcfDNA sequencing only, reference test only, or both)]*

**Table 3b: Plasma mcfDNA sequencing compared to composite reference standard at any mcfDNA detection level**

|  |  | **Composite reference standard**  (blood culture, pleural fluid culture, and species-specific pleural fluid PCRs targeting *Streptococcus pneumoniae,* group A *Streptococcus,* and *Staphylococcus aureus*) | |  |
| --- | --- | --- | --- | --- |
| **Plasma mcfDNA sequencing**  (any MPM) |  | **+** | **−** | **Total** |
|  | **+** | 34 | 9^a^ | 43 |
|  | **−** | 3^b^ | 5 | 8 |
| **Total** |  | 37 | 14 | 51^c^ |

^a^ 9 probable pathogens (across 7 patients) detected by plasma mcfDNA sequencing only were *Streptococcus intermedius* (3), *Fusobacterium nucleatum* (2), *Haemophilus influenzae* (2), *Streptococcus pneumoniae* (1), and *Mycoplasma pneumoniae* (1)

^b^ 3 probable pathogens (across 3 patients) detected by composite reference standard only were *Streptococcus intermedius* (1), *Prevotella* (1), and *Moraxella catarrhalis* (1)

^c^ Excludes 4 probable pathogens (across 3 patients) due to incomplete composite reference standard (missing blood cultures)

**Positive percent agreement** = 91.9% (83.1% - 100.0%)

**Negative percent agreement** = 35.7% (10.6% - 60.8%)

**Jaccard index** = 0.74

**Table 3c: Plasma mcfDNA sequencing compared to culture-based testing at any mcfDNA detection**

|  |  | **Blood and pleural fluid cultures** | |  |
| --- | --- | --- | --- | --- |
| **Plasma mcfDNA sequencing**  (any MPM) |  | **+** | **−** | **Total** |
|  | **+** | 9 | 34^a^ | 43 |
|  | **−** | 3^b^ | 5 | 8 |
| **Total** |  | 12 | 39 | 51^c^ |

^a^ 34 probable pathogens (across 32 patients) detected by plasma mcfDNA sequencing only were *Streptococcus pneumoniae* (15), group A *Streptococcus* (10), *Streptococcus intermedius* (3), *Fusobacterium nucleatum* (2), *Haemophilus influenzae* (2), Methicillin-susceptible *Staphylococcus aureus* (1), and *Mycoplasma pneumoniae* (1)

^b^ 3 probable pathogens (across 3 patients) detected by cultures only were *Streptococcus intermedius* (1), *Prevotella* (1), and *Moraxella catarrhalis* (1)

^c^ Excludes 4 probable pathogens (across 3 patients) due to incomplete cultures (missing blood cultures)

**Positive percent agreement** = 75.0% (50.5% - 99.5%)

**Negative percent agreement** = 12.8% (2.3% - 23.3%)

**Jaccard index** = 0.20

**Table 3d: Plasma mcfDNA sequencing compared to composite reference standard at mcfDNA levels > 300 MPM**

|  |  | **Composite reference standard**  (blood culture, pleural fluid culture, and species-specific pleural fluid PCRs targeting *Streptococcus pneumoniae,* group A *Streptococcus,* and *Staphylococcus aureus*) | |  |
| --- | --- | --- | --- | --- |
| **Plasma mcfDNA sequencing**  (MPM >300) |  | **+** | **−** | **Total** |
|  | **+** | 32 | 8 | 40 |
|  | **−** | 4 | 5 | 9 |
| **Total** |  | 36 | 13 | 49^a^ |

^a^ Excludes 6 probable pathogens (across 5 patients); 4 excluded due to incomplete composite reference standard (missing blood cultures) and 2 excluded due to non-quantifiable mcfDNA levels

**Positive percent agreement** = 88.9% (78.6% - 99.2%)

**Negative percent agreement** = 38.5% (12.0% - 64.9%)

**Jaccard index** = 0.73

**Table 3e: Plasma mcfDNA sequencing compared to culture-based testing for detection of probable cCAP pathogens at mcfDNA levels > 300 MPM**

|  |  | **Blood and pleural fluid cultures** | |  |
| --- | --- | --- | --- | --- |
| **Plasma mcfDNA sequencing**  (MPM >300) |  | **+** | **−** | **Total** |
|  | **+** | 8 | 32 | 40 |
|  | **−** | 3 | 6 | 9 |
| **Total** |  | 11 | 38 | 49^a^ |

^a^ Excludes 6 probable pathogens (across 5 patients); 4 excluded due to incomplete cultures (missing blood cultures) and 2 excluded due to non-quantifiable mcfDNA levels

**Positive percent agreement** = 72.7% (46.4% - 99.1%)

**Negative percent agreement** = 15.8% (4.2% - 27.4%)

**Jaccard index** = 0.19
